# Novel approach to gastric mucosal defect repair using fresh amniotic membrane allograft in dogs (experimental study)

**DOI:** 10.1186/s13287-017-0682-3

**Published:** 2017-10-18

**Authors:** Haithem A. Farghali, Naglaa A. AbdElKader, Marwa S. Khattab, Huda O. AbuBakr

**Affiliations:** 1 Department of Surgery, Anesthesiology, and Radiology, Faculty of Veterinary Medicine, Cairo University, Giza, 12211 Egypt; 2 Department of Pathology, Faculty of Veterinary Medicine, Cairo University, Giza, 12211 Egypt; 3 Department of Biochemistry and Chemistry of Nutrition, Faculty of Veterinary Medicine, Cairo University, Giza, 12211 Egypt

**Keywords:** Gastric mucosa, Amniotic membrane, Allograft, Endoscope, Pepsin, Immunohistochemistry

## Abstract

**Background:**

Gastric mucosal defect could result from several causative factors including the use of nonsteroidal anti-inflammatory drugs, *Helicobacter pylori* infection, gastrointestinal and spinal cord diseases, and neoplasia. This study was performed to achieve a novel simple, inexpensive, and effective surgical technique for the repair of gastric mucosal defect.

**Methods:**

Six adult male mongrel dogs were divided into two groups (three dogs each). In the control positive group (C + ve), dogs were subjected to surgical induction of gastric mucosal defect and then treated using traditional medicinal treatment for such a condition. In the amniotic membrane (AM) group, dogs were subjected to the same operation and then fresh AM allograft was applied. Clinical, endoscopic, biochemical (serum protein and lipid and pepsin activity in gastric juice), histopathological, and immunohistochemistry evaluations were performed.

**Results:**

Regarding endoscopic examination, there was no sign of inflammatory reaction around the grafted area in the AM group compared to the C + ve group. The leukocytic infiltration in the gastric ulcer was well detected in the control group and was less observed in the AM group. In the AM group, the concentrations of both protein and lipid profiles were nearly the same as those in serum samples taken preoperatively at zero time, which indicated that the AM grafting acted the same as gastric mucosa. The re-epithelization of the gastric ulcer in the C + ve group was not yet detected at 21 days, while in the AM group it was well observed covering most of the gastric ulcer. AM accelerated the re-epithelization of the gastric ulcer. The fibrous connective tissue and the precursor of collagen (COL IA1) were poorly detected in the gastric ulcer with AM application.

**Conclusion:**

Using fresh AM allograft for repairing gastric mucosal defect in dogs showed great impact as a novel method to achieve optimum reconstruction of the gastric mucosal architecture and restoration of pre-epithelial, epithelial, and post-epithelial normal gastric mucosal barriers.

## Background

The gastric mucosal barrier is a complex system which involves physical, chemical, and biological defense mechanisms that protect the stomach against irritant ingested food, hydrochloric acid, and excessive pepsin activity. Several conditions such as gastritis, gastric erosions, and ulceration can disrupt the gastric mucosal barrier leading to its damage [1]. Gastric mucosal injuries are common in veterinary medicine because of many regularly used drugs such as NSAIDs or glucocorticoids. There are several diseases that can overwhelm mucosal defense mechanisms including *Helicobacter pylori* infection, hepatic or renal diseases, hypoadrenocorticism, shock, spinal cord disease, autoimmune conditions, primary gastrointestinal disease, and neoplasia [1, 2]. Therefore, gastric or duodenal ulcers usually result from a defect in barrier function of either the gastric mucosa or the duodenal epithelium. Gastric ulceration has been described as grossly detectable defects in the gastric mucosa appearing as a single, large “moon crater” defect [3].

Endoscope studies revealed that 48.5% of canine athletes suffer from ulceration in the stomach or the proximal duodenum. Additionally, gastric cancer is also prevalent in canines compared to other domestic animals in which tumor resection of gastric cancers would result in deep gastric wounds that require tissue reconstruction [4]. The majority of gastric malignancies in dogs are carcinomas, accounting for 50–90%, followed by leiomyosarcomas and malignant lymphoma [5, 6] which is likely similar to the etiology and pathogenesis of human tumors [7, 8].

Several studies have sought to graft gastric mucosal defect. One promising novel biological material that could be used is amniotic membrane (AM). AM is the innermost layer of the fetal membranes and comprises a single layer of epithelial cells on a thicker basement membrane besides spongy collagen IV, V, and VII in addition to a fibronectin and laminin layer containing mesenchymal cells [9]. AM is considered a suitable and excellent tissue for allograft based on its low immunogenicity. There is no risk of rejection as amnion surface cells do not express HLA A, HLA B, HLA C, or β2-microglobulin [10, 11]. Moreover, AM has the ability to suppress T lymphocytes in allografted limbal cells [12]. AM serves as a basement membrane that facilitates epithelial cell migration, reinforces adhesion of basal epithelial cells [13], promotes epithelial differentiation [14], and prevents epithelial apoptosis [15]. Furthermore, amniotic cells release physiological levels of cytokines relevant to wound healing, including platelet-derived growth factor, vascular endothelial growth factor, angiogenin, transforming growth factor beta 2 (TGF-β2), tissue inhibitor of metalloproteinase 1 (TIMP-1), and TIMP-2 [16].

AM also possesses anti-inflammatory, anti-fibrotic, anti-angiogenic, and antimicrobial properties. It inhibits fibrosis as it induces downregulation of TGF-β signaling responsible for fibroblastic activation in wound healing. Application of AM on wounds also results in significant pain relief in burns, due to adhesion to the wound surface and coverage of the dermal nerve endings [17]. Many studies have demonstrated that cells derived from AM are able to differentiate into many kinds of mature cells, including adipocytes, osteocytes, chondrocytes, myocytes, cardiomyocytes, hepatocytes, neurocytes, and vascular endothelial cells. These observations suggest that AM contains stem cell-like cells and could, therefore, provide an alternative source of cells for regenerative medicine [18].

AM has been used for nearly a century in reconstructive surgery. It was used on burned and ulcerated skin, as a biological dressing for open wounds, as a graft in ophthalmic surgery, for reconstruction of the oral cavity and bladder, as a neo-vaginal graft, and for tympanoplasty and arthroplasty [17, 19].

To date, most clinical experiences with human AM transplantation were with tissue preserved in glycerol solution or by cryopreservation [20, 21]. Recent studies suggest that amniotic epithelial cells are not viable after preservation and it is unclear whether the growth factors survive by cryopreservation. Several surgeons have described the use of the fresh human AM for transplantation in vaginal prolapsed repairs with no serious complications [22, 23].

The objective of this study is to describe a simple, novel, inexpensive, and effective surgical technique for gastric mucosal defect repair in dogs using a fresh AM allograft.

## Methods

### Animals

This study was approved by the Animal Use and Care Committee at Faculty of Veterinary Medicine, Cairo University, Egypt. All surgeries were carried out under general anesthesia, and all efforts were made to minimize animal suffering and to reduce the number of animals used. A total number of six adult male mongrel dogs aged approximately 3–5 years and weighing 20–25 kg were used in the present study. The animals were divided into two groups (three dogs each). In the AM group, dogs were subjected to surgical induction of gastric mucosal defect and then treated using fresh AM allograft. In the control positive (C + ve) group, dogs were subjected to the same operation and then treated using traditional medicinal treatment for such a condition. The animals were kept in kennels at the experimental unit of the Department of Surgery, Anesthesiology and Radiology, Faculty of Veterinary Medicine, Cairo University under standard environmental conditions (23 ± 1 °C, with 55 ± 5% humidity and a 12-h light/dark cycle) and maintained with free access to water and fed a maintenance ration twice daily.

### Preparation of amniotic membrane

AM was collected from fresh specimen placenta delivered by elective cesarean section with an intact membrane. It was then rinsed with sterile saline solution to remove any debris after peeling off the chorion. Under aseptic measures, the placenta was washed thoroughly with sterile normal saline, containing 100 U/ml penicillin and 0.2 mg/ml streptomycin (Pen & Strept; Norbrook, the Netherlands) and 0.025 mg/ml amphotericin B. The membrane was then rinsed several times with normal saline mixture in a sterile plastic Petri dish marking the epithelial side with mersilk 4/0 suturing material. Following that, AM was placed in a Petri dish containing the same mixture, and then stored in a refrigerator at 4 °C to be used within a week [21, 24].

### Induction of mucosal defect in stomach and amniotic membrane graft

**Fig. 1:**
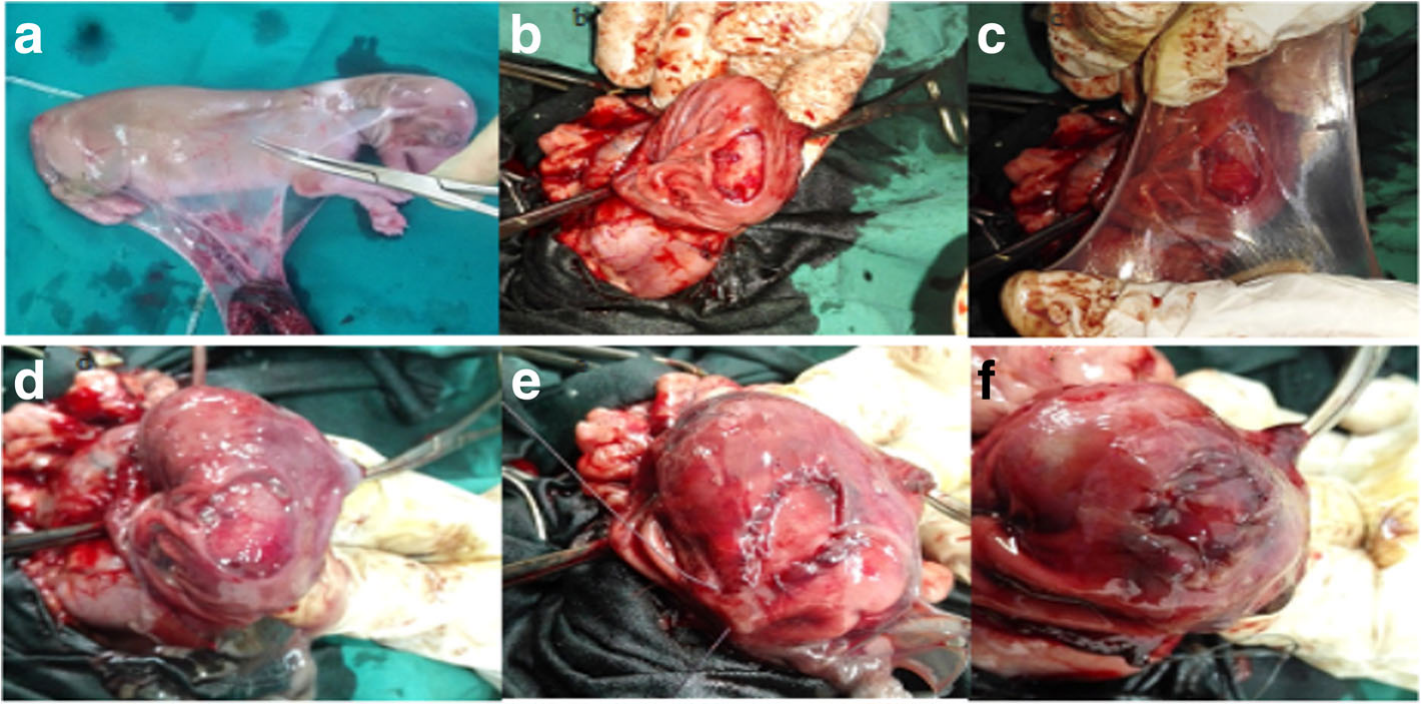
Collecting, harvesting, and grafting AM. **a** Collected AM (innermost transparent and thin fetal membrane). **b** Surgical induction of circular gastric mucosal defect. **c** Application of AM with epithelial lining side up. **d** AM covering gastric mucosal defect. **e** Suturing the first layer with simple continuous. **f** Trimming the AM after suturing the second layer

Under general injectable anesthesia, each dog was premedicated with atropine sulphate (1%®, 0.05–0.1 mg/kg b.wt.; AdwiaCo. S.A.E., Egypt) and xylazine (Xyla-Ject 2%®, 1 mg/kg b.wt.; AdwiaCo. S.A.E.), and then anesthesia was induced using ketamine HCl (Ketalar®, 10–15 mg/kg b.wt.; Sigma-Tec, Egypt) and maintained by ketamine HCl [16, 25] under complete aseptic conditions. The selected dogs were subjected to laparo-surgeries in which the ventral midline approach was adopted and a 3 cm × 2 cm circular patch in the mucosal surface of a body part of an empty stomach was resected. In the AM group, the induced ulcer was replaced by double layers of 4 cm × 5 cm fresh AMs (“zone of altered morphology” (ZAM) is not preferred). The first layer was sutured to the gastric mucosal wound with simple continuous sutures using vicryl 3/0 with the epithelial side up, followed by suturing a second layer in the same manner (Fig. 1).

In the C + ve group, a circular patch was made without any coverage. The opposing laparotomy wound layer was sutured using vicryl 2/0. The animals were treated using traditional medicinal treatment for such a condition. They received proton pump inhibitor drugs (Pepazol®, omperazol 40 mg b.i.d.) and mucosal coater (mucogel®, t.i.d.).

### Postoperative care

Food was withheld for the first 3 days to avoid stimulation of gastric acid and pepsin secretion, and substitution fluid therapy (dextrose 5% sol and Ringer’s sol) was used. The animals were then fed on boiled rice and chicken. Both the AM and C + ve groups were given flagyl infusion/12 h and flumox 500 mg/12 h for 7 days to avoid helicobacter affections [26]. Wounds were cared for daily and the skin sutures were removed 10 days post operation.

### Clinical evaluation

The animals in this study were subjected to daily clinical examination of general health conditions like body temperature, heart and respiratory rates, mucous membrane color, lymph node size, feeding appetite, and urination and defecation episodes and character.

### Endoscopic evaluation

Each dog in both the AM and C + ve groups was anesthetized after 10 and 21 days using the previous general injectable anesthetic regime. The endoscopic images for the stomach of the examined dogs were captured using the Eickemeyer video-endoscope unit supplied (3-m length and 2.3-mm working channel).

### Sampling

#### Blood samples

Blood samples were collected from the jugular vein; a portion was stored on EDTA, and the other portion was left to clot in clear dry centrifuge tubes and then centrifuged at 3500 rpm for 15 min.

#### Gastric juice

From the two groups, samples were collected parallel to endoscopic evaluations through catheterization.

Both samples were collected before the operation and 10 and 21 days after the operation, and were stored at –80 °C for biochemical and pepsin concentration analysis.

#### Surgical biopsies

Gastrostomy was performed to collect surgical biopsies at the 21st postoperative day from the AM and C + ve groups for histopathological and immunohistochemical evaluation.

### Hematological analysis

#### Complete blood pictures

The automated system (Abacus 380; Diatron) was used for analyzing blood parameters and comparing to reference ranges [27].

#### Leukogram

The differential leukocytic count was analyzed by the traditional method as follows: a drop of blood was thinly spread over a glass slide. The blood film was fixed with methyl alcohol for 2 min, stained with diluted Giemsa stain 1:9 with buffer for 8–10 min, and then the smear was washed off with buffer and dried.

Under the oil immersion objective, an area where the morphology of the cells is clearly visible was chosen by moving the slide in the area including the center and periphery of the smear. A total of 200 cells were counted in which every white cell seen was recorded [28].

### Biochemical analysis

Total protein and albumin levels were estimated according to the methods described by Doumas [29]. Serum globulin was calculated according to Lanter [30]. The serum triglyceride concentration was determined according to the method of Fossati and Prencipe [31] and the serum cholesterol concentration was estimated according to the method of Deeg and Ziegenohrm [32] using reagent kits purchased from Spectra Company (Egypt).

#### Evaluation of pepsin activity in gastric juice

The determination of pepsin activity of gastric juice was performed using casein as a substrate according to the method described by Hawk et al. [33]. One milliliter from various concentrations of bovine pepsin, ranging from 0.1 to 1.0 mg/100 ml in 0.1 N HCl, was transferred to a test tube and incubated for 30 min with 3.9 ml of the substrate in a water bath at 37 °C. Then, 10 ml of TCA was added and the tubes were left standing for 10 min and filtered. Blanks were made for each concentration by adding 10 ml of TCA before the addition of the enzyme. The optical density of the filtrate was measured at 280-nm wavelength. For the determination of the proteolytic activity of gastric secretion, the same procedure was followed at a concentration of 2% of 0.1 N HCl.

#### Histopathological evaluation

Biopsies from control positive and grafted gastric defect were collected at the end of the experiment and fixed in 10% neutral buffered formalin for 48 h. These biopsies were then processed by the paraffin-embedding technique. Tissue sections 5 μm thick were prepared using microtome (Leica 2135) and stained with H&E stain for microscopic examination [34]. Re-epithelization and leukocytic cell infiltrate in induced ulcers were evaluated in control and AM-treated ulcers. Semiquantitative scores of lymphocytes from 0 to 3 were used for inflammation. Inflammation of the ulcerated region was scored by counting lymphocytes in three fields (200× magnification) in three observer-randomized H&E slides (semiquantitative score: 0, < 5% cells/field; 1, 5–25%; 2, 25–50%; 3, > 50%) [35].

Tissue sections from each paraffin-embedded block were also stained with Masson’s Trichrome to detect fibrosis [34]. The stained tissue sections were examined by light microscopy and photographed using an Olympus XC30 camera (Tokyo, Japan).

#### Immunohistochemical evaluation

Collagen I alpha 1 was immunohistochemically stained in paraffin-embedded tissue sections (5 μm thick). After deparaffinization and rehydration of tissue sections, immunohistochemical (IHC) staining was carried out using a primary antibody against collagen IA1 (Novus Biologicals, Europe) prepared in rabbits and the avidin–biotin–peroxidase complex method (Dako, LSAB + system-HRP; North America, Inc.) [34]. All procedures were performed according to the manufacturer’s protocol. Color development was carried out using DAB reagent and hematoxylin was used as a counterstain. Finally, the slides were dried, mounted with Canada balsam, covered, and examined using a light microscope.

### Statistical analysis

All data were statistically analyzed using one-way analysis of variance (ANOVA) followed by LSD and Duncan’s test. All data were expressed as means ± SE. *p* < 0.05 indicated statistical significance. All of the statistical analyses were performed using SPSS Statistics for Windows, version 20 (IBM, Armonk, NY, USA).

## Results

### Clinical findings

The animals under study showed no disturbances of general health parameters like body temperature, heart and respiratory rates, mucous membrane color, lymph node size, feeding appetite, and urination and defecation episodes and character in daily clinical examination all over the experimental period.

### Endoscopic and surgical exploration findings

**Fig. 2:**
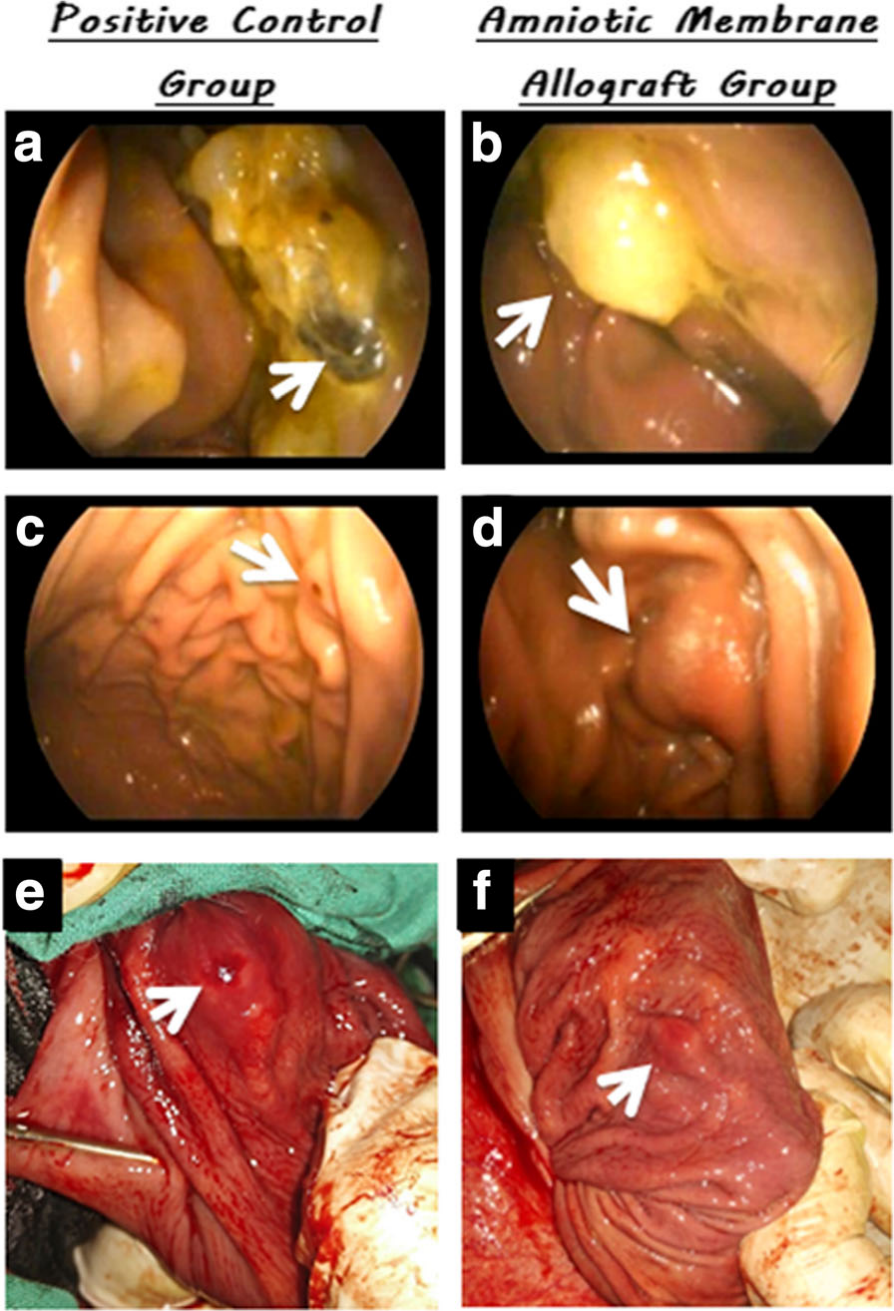
Gastric endoscopic examination at the 10th and 21st postoperative day and exploration findings (after gastrostomy) at 21 days. **a** Gastroscopy of the positive control group at 10th postoperative day; in which the arrow showing clotted blood covering mucosal defect and adhered food remnants. **b** Gastroscopy of the AM group at 10th postoperative day; the arrow revealing AM covering mucosal defect with neither inflammatory reaction nor bleeding in surrounding mucosa. **c** Gastroscopy of the positive control group at 21st postoperative day; the arrow showing small circular ulceration at the mucosa. **d** Gastroscopy of the AM group at 21st postoperative day; the arrow revealing no detectable mucosal defect or AM remnant. **e** Gastric exploration during surgical biopsy of the positive control group at 21st postoperative day; in which the arrow showing small circular ulceration of gastric mucosa with mild bleeding and surrounded by inflammatory zone. **f** Gastric exploration during surgical biopsy of the AM group at 21st postoperative day; the arrow showing no detectable mucosal defect or AM remnants on the mucosal surface and no signs of inflammation or bleeding

Gastric endoscopic examination at the 10th postoperative day showed clotted blood covering the mucosal defect in the C + ve group with adhered food remnants (Fig. 2a). In the AM group, amniotic membrane covered the mucosal defect with neither inflammatory reaction nor bleeding in surrounding mucosa (appear as part of mucosa) (Fig. 2b). Follow-up using gastric endoscopic examination at the 21st postoperative day in the C + ve group showed small circular ulceration at the mucosa with mild bleeding that was surrounded by an inflammatory zone (Fig. 2c). Regarding the AM group, no identical mucosal defect was detected which nearly disappeared with no detectable AM on the mucosal surface and without any signs of inflammation or bleeding (Fig. 2d).

Gastric exploration during surgical biopsy of the positive control group at the 21st postoperative day showed small circular ulceration of gastric mucosa with mild bleeding surrounded by an inflammatory zone (Fig. 2e). Exploration during surgical biopsy of the AM group at the 21st postoperative day showed no detectable mucosal defect or AM remnants on the mucosal surface with no signs of inflammation or bleeding (Fig. 2f).

### Hematological findings

The complete blood picture revealed a mild decrease in RBCs of different degrees in the control group at 10 and 21 days after the experiment, while the AM group was in the normal average limit at 10 and 21 days. HGB in the control group was severely decreased indicating anemia, but was in the normal average limit in the AM group (Table 1). On the other hand, the leukogram revealed that there were no differences from normal ranges in both the control and treated groups.

**Table 1:** Complete blood picture

Group	WBC	RBC	HGB	HCT	PLT	MCV	MCH	MCHC
C + ve 10 days	6983.33 ± 72.64	4.033 ± 0.08	8.6 ± 0.30	28.70 ± 1.17	310 ± 15.27	71.11 ± 1.35	21.31 ± 0.43	29.98 ± 0.72
C + ve 21 days	7250 ± 132.2	4.43 ± 0.22	9.33 ± 0.29	30.75 ± 1.374	340.33 ± 19.16	69.23 ± 0.67	21.1 ± 0.55	30.36 ± 0.56
AM 10 days	11,972.67 ± 2028.80	5.663 ± 0.375	12.34 ± 0.49	39.40 ± 0.871	342 ± 16.653	69.99 ± 3.20	21.87 ± 0.59	31.30 ± 0.56
AM 21 days	15,023.33 ± 261.1726	6.88 ± 0.04	14.56 ± 0.14	44.87 ± 0.55	365.66 ± 8.950	65.13 ± 0.72	21.16 ± 0.08	32.46 ± 0.38
Reference range	6000–15,000	5.5–8.5	12–18	39–55%	200,000–500,000	60–77	19.1–26.2	32–36

The control group showed a slight decrease in RBC count, while the AM group was in normal average limits

HGB in control is severely decreased, but the AM group was in normal average limits

*AM* amniotic membrane, *C + ve* control positive, *HCT* hematocrit, *HGB* hemoglobin, *PLT* platelet, *RBC* red blood cell, *WBC* white blood cell, *MCV* mean cell volume, *MCH* mean cell haemoglobin, *MCHC* mean cell haemoglobin concentration

### Biochemical findings

**Fig. 3:**
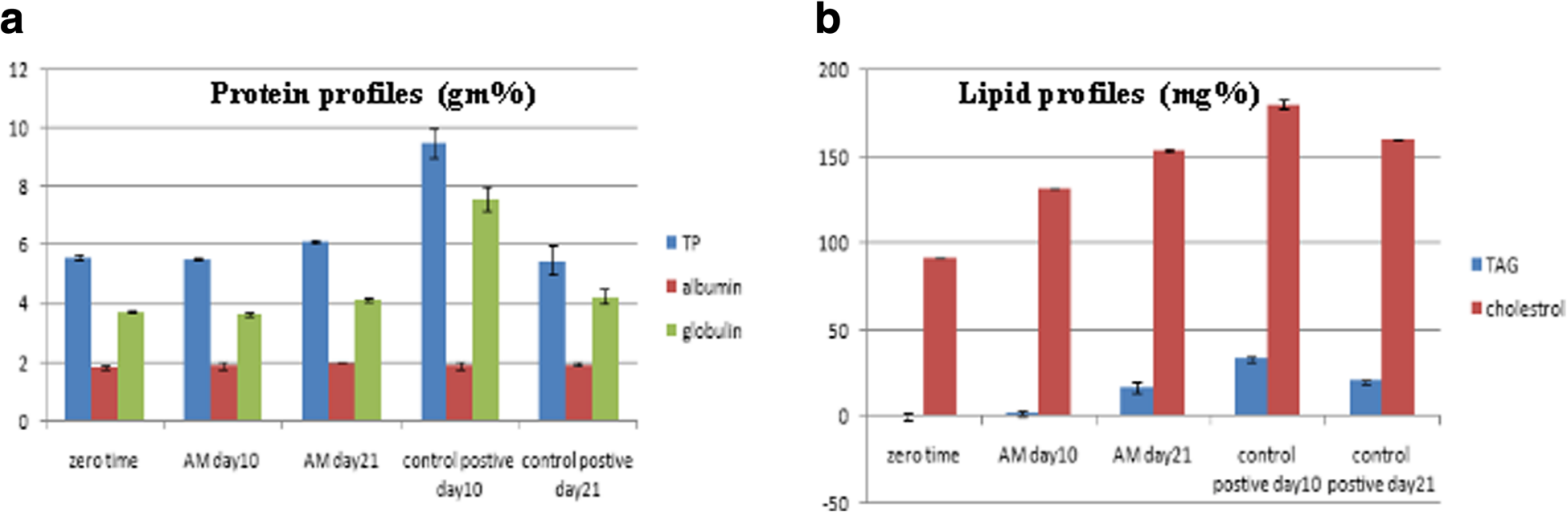
Biochemical findings in AM and C + ve groups at days 0, 10, and 21. **a** Serum protein profiles (g %): concentration of serum TP and globulin concentration significantly increased at C + ve day 10 compared to zero time and the AM group. **b** Lipid (mg %) profiles: serum concentration of TAG and cholesterol significantly increased at C + ve day 10 postoperative compared to zero time and the AM group followed by a gradual decrease within 21 days to be the same as serum of 21 days for the AM group. *AM* amniotic membrane, *TP* total protein, *TAG* triacylglycerol

The concentration of serum total protein (gm%) as well as the globulin concentration (gm%) significantly increased (*p* ≤ 0.05) at the 10th postoperative day in the C + ve group compared to zero time and the AM group (Fig. 3a). Moreover, the serum concentration of TAG and cholesterol (mg%) significantly increased at the 10^th^ postoperative day in the C + ve group compared to zero time and the AM group, followed by a gradual decrease within 21 days to be the same as serum of 21 days for the AM group (Fig. 3b).

### Pepsin activity in gastric juice

**Fig. 4:**
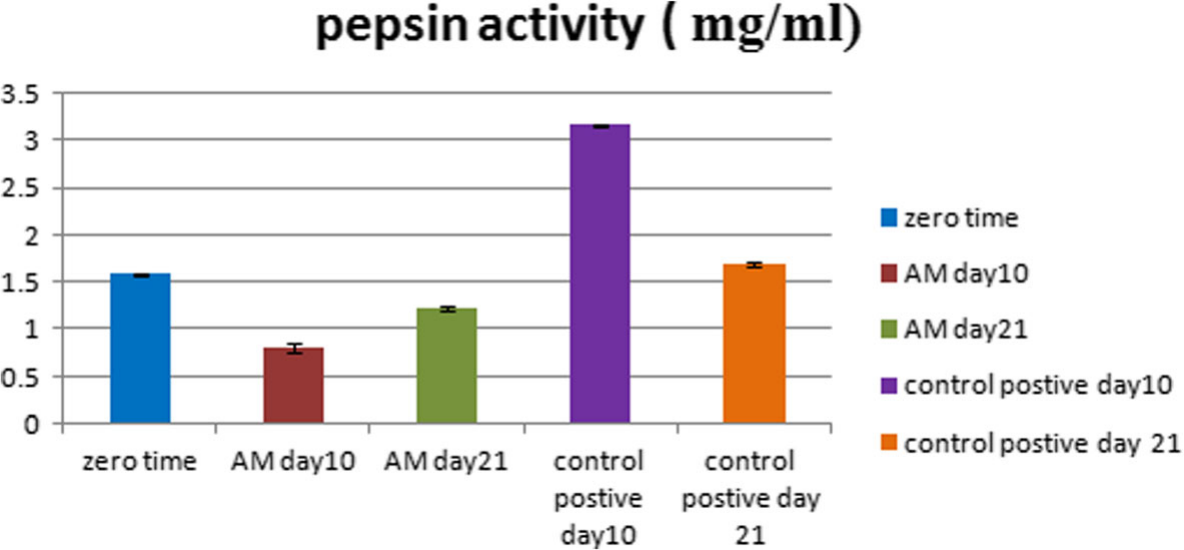
Pepsin activity (mg/ml) in gastric juice in the AM and C + ve groups at days 0, 10, and 21. Activity of pepsin significantly increased in the C + ve group on day 10 and significantly decreased in the AM group on days 10 and 21. AM amniotic membrane

The activity of pepsin was significantly increased on day 10 for the C + ve group (3.165 mg/ml) and significantly decreased in the AM group at days 10 and 21 (0.8 and 1.225 mg/ml respectively) (Fig. 4).

### Histopathological and immunohistochemical findings

**Fig. 5:**
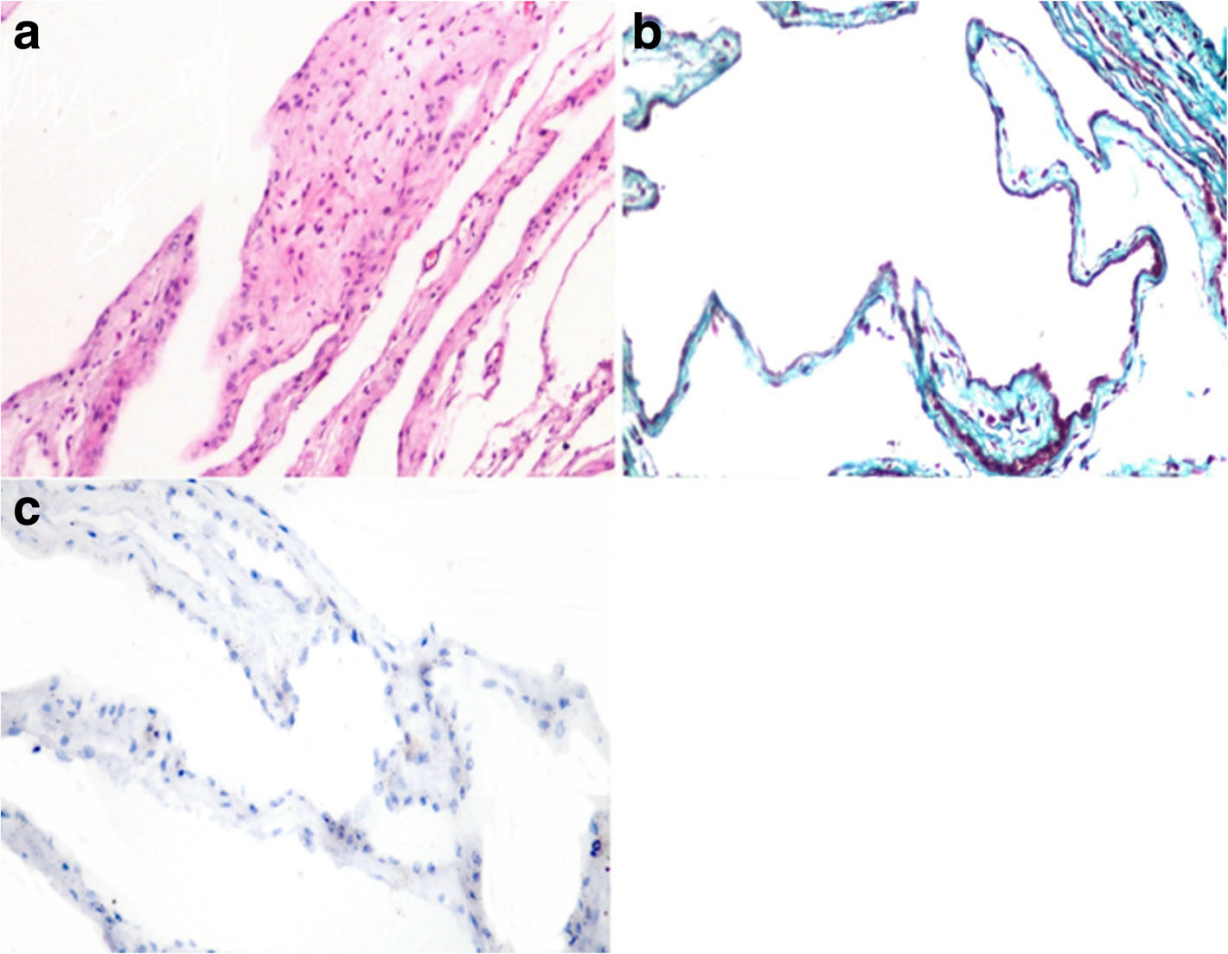
Fresh AM, bitch. **a** Stained with H&E, histopathological examination of fresh AM revealing normal histological structure with presence of surface epithelium and underlying loose connective tissue. **b** Stained with MTC, loose connective tissue of AM stained blue and the fibroblasts stained red. **c** Stained with collagen IA1 (×400), negative staining for collagen IA1 in AM

The histopathological examination of fresh AM revealed normal histological structure with the presence of surface epithelium and underlying loose connective tissue (Fig. 5a). Using MTC stain, the loose connective tissue of the AM stained blue and the fibroblasts stained red (Fig. 5b). There was negative staining for collagen IA1 in the AM (Fig. 5c).

**Fig. 6:**
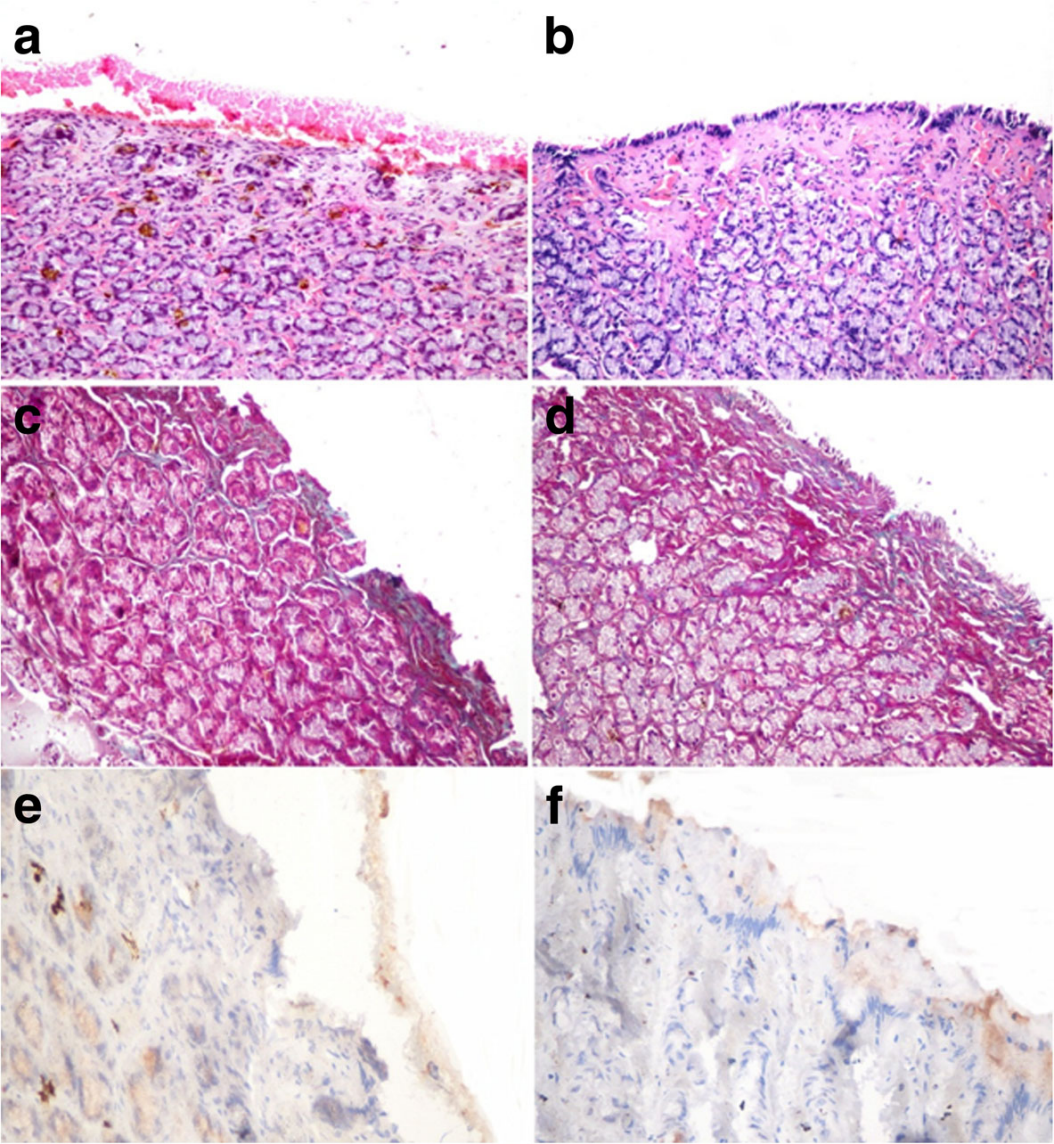
Gastric mucosal defect, dog. **a** Absence of lining epithelium, presence of bleeding ulcer, and mononuclear inflammatory cell infiltration in periglandular connective tissue in the control group. **b** Regenerated lining epithelium, hypervascularization of underlying tissue, and presence of mononuclear inflammatory cell infiltration in periglandular connective tissue (H&E × 200). **c** Poorly observed bluish stained connective tissue in mucosa of control ulcer. **d** Fine fibers of connective tissue below regenerated epithelium in the gastric ulcer with AM graft (Masson’s trichrome × 200). **e** Negative staining of collagen IA1 in the exposed granulation tissue in the control ulcer and **f** negative staining of subepithelial connective tissue in the gastric ulcer with AM graft (avidin–biotin–peroxidase complex method, hematoxylin counterstain × 400)

In the C + ve group, the gastric mucosal epithelium was not yet regenerated leaving a bleeding ulcer. Moreover, mononuclear inflammatory cells were observed in the periglandular connective tissue (2.33 ± 0.33 cells) (Fig. 6a). On the other hand, the gastric ulcer in the group with AM application showed almost complete regeneration of the gastric epithelium with the exception of minute areas that were not yet covered by epithelium (Fig. 6b). Hypervascularization of underlying granulation tissue was well detected in addition to little mononuclear inflammatory cell infiltration in the periglandular connective tissue (1 ± 0 cells).

Using MTC, fine fibers of connective tissue were observed below the regenerated epithelium in the gastric ulcer with the AM graft and in the control gastric ulcer (Fig. 6c, d).

In the control gastric ulcer, there was negative staining of collagen IA1 in the exposed granulation tissue and there were a few areas of weak staining on surface epithelium (Fig. 6e, f).

## Discussion

The challenge facing the authors of the present study was to achieve optimum reconstruction of the gastric mucosal architecture after surgical induction of mucosal defect using fresh AM allograft. Normally, gastric mucosal integrity is maintained by three defense mechanisms; pre-epithelial (mucus–bicarbonate–phospholipid barrier), epithelial (a continuous layer of surface epithelial cells interconnected by tight junctions which generate and secrete bicarbonate, mucus, phospholipids, trefoil peptides, prostaglandins (PGs), and heat shock proteins), and post-epithelial (continuous blood flow through mucosal microvessels lined with endothelial cells forming an endothelial “barrier”, sensory nerves releasing PGs, nitric oxide, and calcitonin gene-related peptide that regulates mucosal blood flow) [36, 37]. The continuity of the epithelial cell layer renewal is maintained by the proliferation of progenitor cells that is regulated by growth factors, prostaglandin E2, and survivin, an antiapoptosis and mitosis-promoting protein [37].

When the integrity of the mucosal barrier is compromised, a cascade of pathologic events contributing to further damage of the mucosal layer takes place. Firstly, the rate of back diffusion of gastric acid and pepsin increases, leading to inflammation and hemorrhage. Besides this, endothelial and inflammatory cells including neutrophils and mast cells become activated and release histamine which promotes further acid secretion, leukotrienes, platelet-activating factor, proteolytic enzymes, and free radicals [38]. These events exacerbate the initial mucosal damage by reducing blood flow, leading to ischemia, impaired cell renewal, and reduced mucus and PG secretion [37, 38].

In our country, the terrible financial constraints of public health and animal care sustainability trigger the continuous discovery of new therapeutic alternatives. One of these therapeutic alternatives is the utilization of “amniotic membrane” (AM) obtained from the placenta which is rich in stem cells [39]. AM is considered an important source and excellent scaffold that easily integrates with host tissue and provides an excellent environment for cell growth and differentiation [11]. It releases physiological levels of cytokines relevant to wound healing, including platelet-derived growth factor, vascular endothelial growth factor, angiogenin, TGF-β2, TIMP-1, and TIMP-2 [11].

The use of AM in repairing tissue defects started during the first half of the last century, in 1910, for skin transplantation, urinary bladders, ocular lesions, burns, varicose ulcers, neovagina reconstruction, nerve damage, mouth sores, and early healing of peritoneal lesions [40–42].

In the present study, during harvesting of AM, the chorion was peeled off to avoid further inflammatory and immunological reaction that might provoke revascularization and inflammatory reaction in the host tissue which sooner or later could result in a rejection phenomenon [21, 43]. In addition, the ZAM (“zone of altered morphology”) was avoided during cutting the graft due to reduce thickness and cellularity of the membrane [11].

The healing of gastric mucosal defect usually requires medical therapy that is directed toward maintaining mucosal perfusion, decreasing gastric acidity, and protecting ulcers [26]. In a recent study, it was proved that AM created the previous referred required healing factors. Firstly, AM maintained mucosal perfusion through the secreted transforming growth factor (TGF) which can stimulate epithelialization and modulate proliferation and differentiation of stromal fibroblasts [44]. Secondly, AM decreased gastric acidity as amniotic epithelial cells (AEC) were able to secrete albumin, which is consistent with α1-antitrypsin and other hepatocyte gene expression profiles which act as a natural neutralizing agent to gastric acid [45]. Thirdly, AM protected the mucosal defect by both mechanical covering and biological protection as AEC produce β-defensins that stimulate a defense mechanism against any microbial infection which could retard the healing process [46].

In our study, we used fresh AM in the treatment of gastric ulcer, although it has been used in the conserved form in different studies [41]. To our knowledge, there are no literature data about gastric ulcer treatment by AM. Therefore we used a fresh form of AM as it is rich in pluripotent stem cells that multiply rapidly, forming a tissue similar to those around it [47]. Besides, epithelial cells derived from AM are clonogenic, and have the advantage of stem cells [48], which resulted in minimizing the time of AM stay in acidic media. Furthermore, fresh AM contains viable AEC and possesses anti-inflammatory properties as it secretes soluble factors that inhibit local activation/migration of neutrophils and macrophages [49–51] and suppress the activation and graft-destroying actions of immune T cells [51, 52]. Moreover, the AM stromal matrix markedly suppresses expression of the potent proinflammatory cytokines IL-1α and IL-1β [11].

**Fig. 7:**
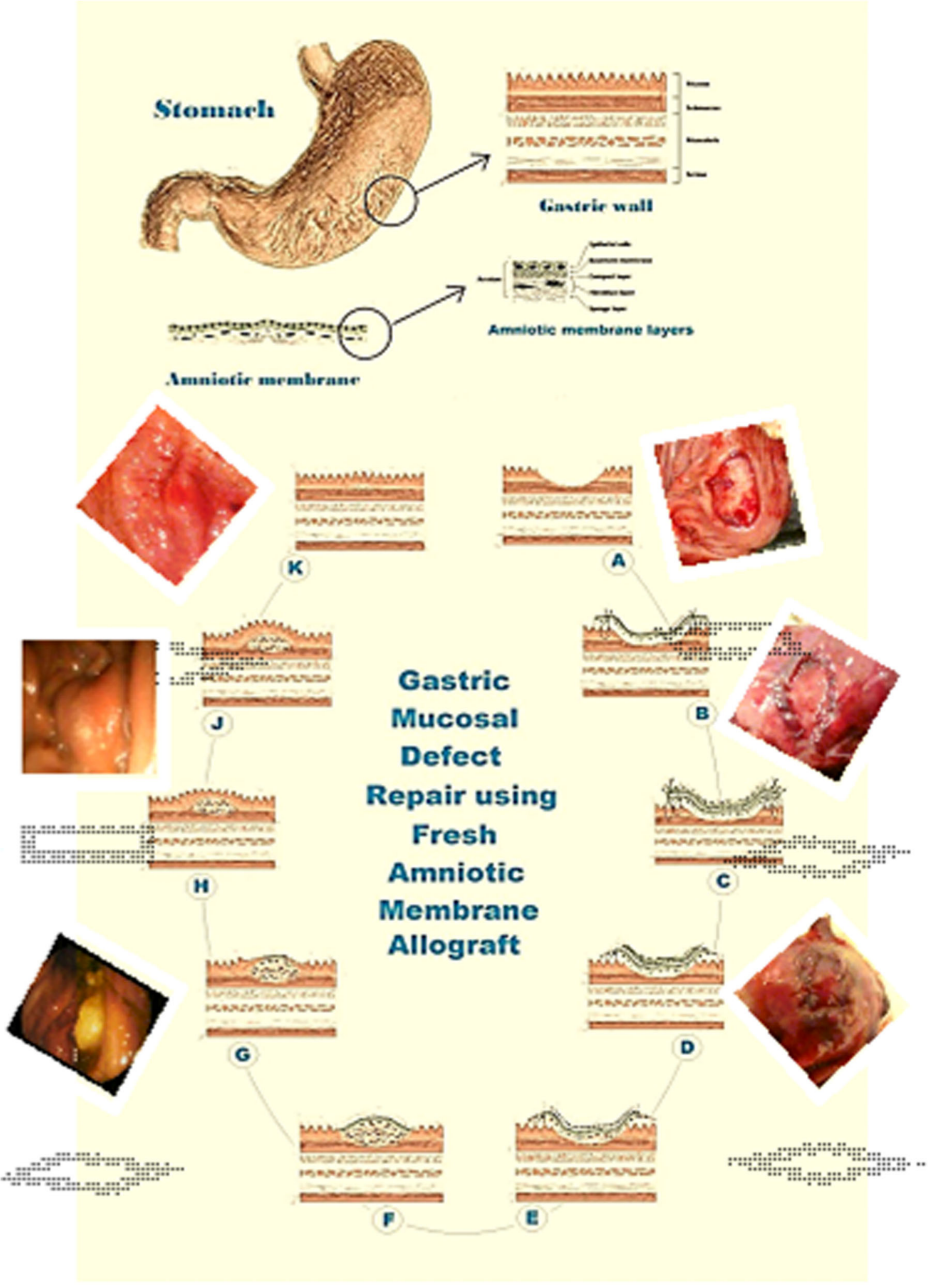
Hypothetical steps of gastric mucosal defect repair using fresh AM allograft. **a** Surgical induction of gastric mucosal defect. **b** Application of AM first layer with the epithelial side up and suturing using a simple continuous suture pattern. **c** Application of AM second layer with the epithelial side down and suturing using a simple continuous suture pattern. **d** Beginning of digestion of the second AM layer. **e** Healing of submucosa was started in addition to continuing of the second AM layer digestion. **f** Complete digestion of the second AM layer and continuing of submucosal healing. **g** Beginning of mucosal cell proliferation and migration from the periphery of the amniotic basement membrane. **h** Complete migration of mucosal cells and continuing of submucosal healing. **i** Well-developed mucosal layer covering the amniotic basement membrane and continuing of submucosal healing. **j** Complete healing of the gastric mucosal defect. **k** Normal reconstitution of gastric mucosal layers

These characteristic features were exploited in our study as proven during endoscopic examination and illustrated in Fig. 7. There was no sign of inflammatory reaction around the grafted area even after 10 days of the experiment in the AM group in comparison to the C + ve group. The leukocytic infiltration in the gastric ulcer was well detected in the control group and was less observed in the AM-treated ulcer. In contrast, Li et al. [53] indicated that the inflammation as seen with animals infected with *H. pylori* usually results in delayed healing of the gastric ulcer. Therefore, application of AM decreased the inflammation in the gastric ulcer which would, in turn, speed up the healing process compared to control ulcers. The AM reducing inflammation was documented previously and was believed to be due to suppressing the expression of the potent proinflammatory cytokines IL-1α and IL-1β by the AM stromal matrix [11].

The re-epithelization of the gastric ulcer in the C + ve group of this study was not yet detected at 21 days compared to the ulcers treated with AM. The re-epithelization in the group treated with AM was not yet complete but was well observed, covering most of the gastric ulcer (Fig. 7). Normally, gastric ulcers heal completely by the 7th week after ulceration [54], and therefore the application of AM has accelerated the re-epithelization of the gastric ulcer.

Sheta et al. [21] and Rodriguez-Ares et al. [55] revealed that preserved AM acted as a splint until the bladder completely healed and sealed itself with the presence of a remnant of the grafted membrane at 1 month, while in the present study there was no remnant of the fresh AM even after 3 weeks. Although further studies are required, this finding might be due to differentiation of the fresh AM cells during the healing process.

In this study, the fibrous connective tissue and the precursor of collagen (COL IA1) were poorly detected in the gastric ulcer with AM application, emphasizing the anti-fibrotic potential of AM owing to the downregulation of TGF-β1, TGF-β2, TGF-β3, and TGG-β receptor II expression in fibroblasts [56].

In the current study, the leukogram revealed a significant difference between groups; however, both groups’ results were still within the normal range. It is important that clinicians do not draw any conclusions from the presence of relative leukocytosis when the total leukocyte count is normal. When laboratories report differential counts, the results are usually accompanied by a normal range, and if the percentage of white cells that are lymphocytes is high then the result may be flagged as abnormal. However, there is no clinical significance to an increase in the percentage of leukocytes in the blood when the total leukocyte count is normal. Usually, such an increase is the result of neutropenia, in which case the cause of the neutropenia should be addressed [57].

In the AM group, the concentrations of both protein and lipid profiles were nearly the same as serum samples taken preoperatively at zero time, which indicates that the AM grafting acted the same as gastric mucosa in which stomach digestion functioned normally. In addition, AM grafting usually accelerates the healing process as a result of the predominance of mature collagen fibers at the early stage of tissue repair [47]. This may explain our result of a significant decrease in the activity of pepsin enzyme in the AM group in comparison to the untreated gastric ulcer. As a result of disruption of gastric epithelial barriers by any means, absorption of HCl across the mucosa and stimulation of the intrinsic nervous system trigger the release of more HCl and pepsin [3]. This mechanism was elucidated in the C + ve group, in which pepsin activity in gastric juice significantly increased at day 10 followed by a significant decrease after 21 days but was not as low as recorded in the AM-treated group.

## Conclusion

From this study it could be concluded that using fresh AM allograft for repairing gastric mucosal defect in dogs showed great impact as a novel method to achieve optimum reconstruction of the gastric mucosal architecture and restoration of pre-epithelial, epithelial, and post-epithelial normal gastric mucosal barriers.

## Abbreviations

AEC: Amniotic epithelial cells
AM: Amniotic membrane
COL IA1: Collagen I alpha 1
NSAID: Nonsteroidal anti-inflammatory drug
PG: Prostaglandin
TCA: Trichloroacetic acid
TGF-β2: Transforming growth factor beta 2
TIMP: Tissue inhibitor of metalloproteinase
